# Implementation science in adolescent healthcare research: an integrative review

**DOI:** 10.1186/s12913-022-07941-3

**Published:** 2022-05-03

**Authors:** Elham Zolfaghari, Natasha Armaghanian, Daniel Waller, Sharon Medlow, Annabelle Hobbs, Lin Perry, Katie Nguyen, Katharine Steinbeck

**Affiliations:** 1 Specialty of Child and Adolescent Health, Faculty of Medicine and Health, The University of Sydney, Sydney, NSW 2006 Australia; 2 Academic Department of Adolescent Medicine, The Children’s Hospital Westmead, Westmead, NSW 2145 Australia; 3 Faculty of Health, University of Technology Sydney, Broadway, Ultimo, NSW 2007 Australia; 4 South East Sydney Local Health District, Prince of Wales Hospital, Randwick, NSW 2316 Australia

**Keywords:** Adolescence, Youth, Health services research, Implementation, Consolidated Framework for Implementation Research, Integrative review

## Abstract

**Background:**

Multiple theories, models and frameworks have been developed to assist implementation of evidence-based practice. However, to date there has been no review of implementation literature specific to adolescent healthcare. This integrative review therefore aimed to determine what implementation science theories, models and frameworks have been applied, what elements of these frameworks have been identified as influential in promoting the implementation and sustainability of service intervention, and to what extent, in what capacity and at what time points has the contribution of adolescent consumer perspectives on evidence implementation been considered.

**Methods:**

An integrative design was used and reported based on a modified form of the PRISMA (2020) checklist. Seven databases were searched for English language primary research which included any implementation science theory, model or framework developed for/with adolescents or applied in relation to adolescent healthcare services within the past 10 years. Content and thematic analysis were applied with the Consolidated Framework for Implementation Research (CFIR) used to frame analysis of the barriers and facilitators to effective implementation of evidence-informed interventions within youth health settings.

**Results:**

From 8717 citations, 13 papers reporting 12 studies were retained. Nine different implementation science theories, frameworks or approaches were applied; six of 12 studies used the CFIR, solely or with other models. All CFIR domains were represented as facilitators and barriers for implementation in included studies. However, there was little or no inclusion of adolescents in the development or review of these initiatives. Only three mentioned youth input, occurring in the pre-implementation or implementation stages.

**Conclusions:**

The few studies found for this review highlight the internationally under-developed nature of this topic. Flagging the importance of the unique characteristics of this particular age group, and of the interventions and strategies to target it, the minimal input of adolescent consumers is cause for concern. Further research is clearly needed and must ensure that youth consumers are engaged from the start and consistently throughout; that their voice is prioritised and not tokenistic; that their contribution is taken seriously. Only then will age-appropriate evidence implementation enable innovations in youth health services to achieve the evidence-based outcomes they offer.

**Trial Registration:**

PROSPERO 2020 CRD42020201142 https://www.crd.york.ac.uk/prospero/display_record.php?RecordID=201142

**Supplementary Information:**

The online version contains supplementary material available at 10.1186/s12913-022-07941-3.

## Contributions to the literature

-
  - Established theories and frameworks are increasingly applied to inform or underpin implementation of changes in adult and paediatric healthcare but this is the first review of their use in adolescent health services research.
-
  - The review found only 13 papers reporting 12 studies; 10 studies originated in North America; the main clinical focus of initiatives was mental health (*n* = 9), also eating disorders (*n* = 1).
-
  - Most factors of the Consolidated Framework for Implementation Research domains were described as implementation facilitators or barriers; age specific determinants of implementation success were only identified when implementation teams considered the characteristics of the target population.
-
  - Consumer engagement was sparse and mostly pre-implementation consultation.

## Introduction

The slow and suboptimal translation of evidence-based practice (EBP) into routine clinical care is a decades-long problem with enduring discrepancies highlighted between the care recommended in evidence-based guidelines and that prescribed and delivered by clinicians or received by patients [1, 2]. Examples have been demonstrated internationally; in Australia, for example, Runciman and colleagues used internationally applied methods to examine 522 indicators of appropriate care for 22 common conditions. In only 57% was care in line with what evidence-based guidelines recommended at the time [3]. Such evidence-practice gaps often result in sub-optimal outcomes for patients and less effective healthcare systems [2].

Multiple factors underpin this evidence-to-practice gap, including unsystematic or under-developed implementation strategies. Recognition of this problem over recent decades has led to development of multiple conceptual theories, models and frameworks to assist implementation of EBP and programs in healthcare. The next steps forward came with consolidation of this body of work into a ‘meta-theoretical’ synthesis of theories, presenting a comprehensive overview of factors shown to be influential across healthcare implementation settings. Building on and updating Greenhalgh’s original work which analysed findings of 495 studies [4], Damschroder et al. mapped the constructs of eighteen published theories into the Consolidated Framework for Implementation Research (CFIR) [5]. Composed of 39 factors organised as five domains (the intervention, inner and outer settings, the individuals involved, and the process by which implementation is accomplished), the CFIR has been extensively used to plan and evaluate implementation strategies and has to date (November 2021) been cited > 2800 times in PubMed and > 7000 times in Google Scholar [6].

With the advent of the CFIR (and implementation science approaches more widely), understanding of how and why implementation strategies succeed or fail has expanded [7]. Structured approaches to implementation planning are now well-established in mainstream service development and quality improvement in adult healthcare (see, for example, in Australia New South Wales Health’s adoption of the Accelerating Implementation Methodology) [8]. However, little is known about the penetration of implementation science frameworks into adolescent healthcare. Adolescent healthcare comprises multiple systems and service transitions where multi-level ecological factors interact interdependently and simultaneously. Implementation efforts within such systems can be overwhelmingly complex due to multiple inputs and influences (e.g. adolescent and family consumers, clinicians, clinical and non-clinical teams and service departments), variations across settings (e.g. paediatric and adult hospitals, community and primary care) and transitions to multiple public and private, governmental and non-governmental services. Arguably, this makes the use of systematic and credible approaches to implementation even more important. It is therefore timely to examine the factors associated with successful evidence implementation in this relatively under researched group to better support practitioners and service providers. Accordingly, this integrative review was designed to describe the current state-of-play for use of implementation science approaches in health services research for adolescents.

### Review Questions

1. What implementation science theories, models and frameworks have been applied in support of service development, innovation or sustainability in adolescent healthcare?
2. What elements of these frameworks have been identified as influential in promoting the implementation and sustainability of service intervention?
3. To what extent and in what capacity has the contribution of adolescent consumer perspectives on evidence implementation been identified or reported in the development and application of implementation frameworks? At what time points were adolescent perspectives considered?

## Methods

An integrative review design was chosen as it was anticipated that studies might use a variety of methods and offer both qualitative and quantitative data. An integrative review has the capacity and flexibility to manage this [9]. Methods were based on and reported in line with a modified form of the PRISMA (2020) recommendations [10].

### Search strategies and screening

Search strategies were developed based on the framework of Participant and Situation [11]:

*Participants* comprised adolescents and youth within the range of 10–25 years (hereafter referred to as adolescents), and their families, as the target group for the proposed intervention. This life stage was chosen as the time when healthcare services need to adjust their interventions to accommodate emerging adolescents’ autonomy and where families’ and carers’ roles are changing. Study participants could also include staff and stakeholders for the proposed intervention.

*Situation:* where any named implementation science theories, frameworks and approaches were used; for this study these were defined as any designated structural arrangement of factors or variables described as influencing or impacting the achievement of behavioural, procedural or service change as a result of intentional effort to integrate research evidence into routine daily practice.

### Inclusion and Exclusion Criteria

To be eligible for inclusion studies were required to:

- Include any implementation science theory, framework or approach that:
  - ◦ Was developed for or with adolescents, or
  - ◦ Was applied in relation to healthcare services designed for delivery to adolescents (adolescents / youth / young people alone or in conjunction with children),
- Be written in the English language,
- Report primary research, and
- Have a publication date within the period January 2010—September 2020.

Papers were excluded where they:

- Did not report findings of primary research studies (e.g. polemic, discussion or protocol papers),
- Were brief reports or abstracts only, including conference abstracts, where full study details were not available,
- Were deemed to focus on services that were not primarily healthcare provider services (e.g., where the intervention was designed or implemented as a public health initiative, was delivered as a school, judicial system or peer-support initiative).

### Literature searching

The search strategy was devised to capture studies that applied any recognised implementation science theory, model or framework for participants across any healthcare specialty. Seven databases were searched: Excepta Medica (Embase), Medical Literature Analysis and Retrieval System Online (Medline), PsycInfo, the Cumulative Index of Nursing and Allied Health Literature (CINAHL), Allied and Contemporary Medicine Database (AMED), the Cochrane Database of Systematic Reviews and the Cochrane Central Register of Controlled Trials. Search strategies were developed consisting of a range of synonyms with abbreviations and wildcards combined with Boolean operands; searches were tailored to each database. Examples of the search strategy are provided in Supplementary File 1. The reference lists of included studies and reviews were also searched for relevant papers.

Search output was downloaded to Endnote version X9. Manual screening and elimination of duplicates was conducted by the first author, then remaining files were uploaded to Covidence [12]. All authors screened the titles and abstracts; every paper was screened independently by two authors. Papers that were clearly ineligible (did not meet inclusion or met exclusion criteria) were removed but in case of uncertainty were retained for full review. Decisions were discussed to agreement; where two reviewers could not agree, a third reviewer adjudicated.

**Fig. 1:**
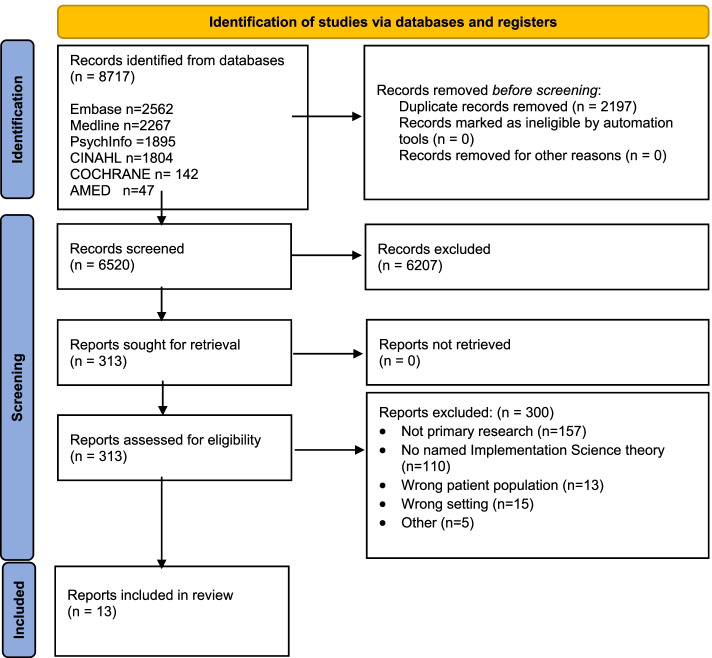
**PRISMA diagram of literature searching and screening***From:* Page MJ, McKenzie JE, Bossuyt PM, Boutron I, Hoffmann TC, Mulrow CD, et al. The PRISMA 2020 statement: an updated guideline for reporting systematic reviews. BMJ 2021;372:n71. https://doi.org/10.1136/bmj.n71 For more information, visit: http://www.prisma-statement.org/

The combined searches produced *n* = 6,520 unique citations. After title and abstract screening, 313 potentially eligible papers were retained for full-text review. Of these, thirteen papers were retained for data extraction (Fig. 1).

### Critical appraisal and data extraction

Papers retained after the screening process were critically appraised using the Mixed Methods Appraisal Tool [13]. Each paper was independently appraised by two authors with conclusions compared and discussed to consensus among the author group. Findings were recorded in Supplementary file 2. No study was excluded on the basis of appraisal findings.

Each article was read independently and core data were extracted into a dedicated spreadsheet by two reviewers across the following categories:

-
  - Study methods and main findings;
-
  - The intervention purpose/ aim, elements (including facilitators and barriers) and target (e.g. patients/ clients);
-
  - The implementation process, comprising implementation science theory/ model/ frameworks named and applied in the study, the implementation target population, and planning, preparation, delivery, monitoring and evaluation activities; any adolescent involvement in preparation, implementation or evaluation.

Data were reviewed with discrepancies discussed and resolved to consensus at author group meetings.

## Data Analysis

Data from included papers were summarised and synthesised using a combination of content and thematic analysis [14] to address the review questions. Content analysis was used to tabulate and summarise data in response to research questions one and three. For research question two, thematic analysis was employed with a combination of inductive and deductive approaches using the CFIR as an organising framework. Each paper was initially coded independently by two authors against CFIR factors, and subsequently discussed to agreement by the author group. Findings were then synthesized and summarised by the third and sixth authors. The CFIR was chosen because it was developed to integrate and unify multiple prior Implementation Science theories, many of which addressed only specific aspects of implementation, featured overlapping constructs and used inconsistent definitions and terminology. The CFIR posits that five domains influence implementation. The intervention itself is one, and salient characteristics include its underpinning empirical evidence and level of complexity. The outer and inner settings have been defined as (outer setting) ‘the economic, political, and social context within which an organization resides’, and (inner setting) ‘features of structural, political, and cultural contexts through which the implementation process will proceed’[5]. The outer setting includes, for example, consumer needs and the availability of resources, policies and incentives whilst the features of the implementation site comprise the inner setting. The characteristics of the individuals involved comprise another domain (e.g. their knowledge and motivation for change) and, lastly, the chosen implementation processes (e.g. activities undertaken in planning, engagement, execution and evaluation) [5]. The purpose of using the CFIR was to demonstrate the barriers and facilitators to effective implementation of evidence-informed interventions within adolescent health settings. Additionally, evidence was sought for any other factors, specifically those that might be unique to adolescent healthcare.

## Results

### Study selection

Figure 1 summarises the literature searching and screening process. From the originally downloaded 8,717 citations, 13 papers were retained for inclusion in the integrative review (Table 1). Of these, two papers related to different stages of implementation within the same study; both were retained. Of the twelve studies, five each were conducted in the USA and Canada, and one each in the UK and Sweden. Nine related to health services for mental health and one to eating disorders; one each related to disability and chronic illness services. Included studies used quantitative, qualitative, and mixed methods study designs in three, four and five studies respectively.

**Table 1:** Characteristics of included papers

Author, year, country	Topic area	Recruitment setting	Timepoint	Study design	Study aims	Study participants	Implementation: Target population
Amaya-Jackson et al., 2018, USA [26]	Mental health	Rural, underserved geographic regions (North Carolina)	Implementation monitoring	Quantitative descriptive	Evaluation of pilot to examine whether: 1. Clinicians in a community practice setting could implement an EBT (e.g. TF-CBT) with a high level of practice fidelity through participation in a LC 2. Youth who participate in a full course of TF-CBT provided by a clinician trained to model fidelity will experience clinically significant symptom improvements	Clinicians: 124 clinicians in 2 × TF-CBT LCs Clients: 281	Clinicians & child clients
Anaby et al., 2015, Canada [22]	Children and youth with disabilities	Paediatric rehabilitation centre	Post-implementation	Qualitative	1. To identify site-specific needs and issues of clinicians working with children and youth with physical disabilities surrounding the theme of participation and the environment 2. To develop and evaluate an intervention plan to facilitate knowledge uptake by clinicians in the clinical context and, consequently, foster change in practice	2 groups of 7 clinicians of MDTs, *n* = 14. Mainly OTs and PTs, working with clients aged 0–21 years	Children and young adults with disabilities, aged 0–21 years
Beidas et al., 2016, USA [24]	Mental health	14 community mental health clinics across the Philadelphia metropolitan area	Pre-training, post-training	Mixed methods case study	1. Describe the context within which the trauma-informed system and the evaluation were developed 2. Describe the implementation science framework that guides the evaluation 3. Present data with regard to implementation determinants and outcomes. 4. Provide recommendations, based on lessons learned, for developing and evaluating a trauma-informed public behavioral health system that links to other youth-serving systems	TF-CBT therapists: four of six staff training cohorts	TF-CBT therapists
Couturier et al., 2018, Canada [19]	Eating disorders	Academic Health Science Centre, community-based behavioural health provider organisations and paediatric eating disorder programs	Post-implementation	Mixed methods	To identify and describe themes arising in the implementation consultation component of the model	One Academic Health Science Centre, 3 community-based behavioural health provider organisations and 17 medical practitioners and administrators	Medical practitioners and administrators
Couturier et al., 2021, Canada [20]	Eating Disorders	Four sites in Ontario who had behavioural health providers who used Family Based Therapy (FBT) for eating disorders	Pre and post implementation	Mixed methods	Implementation of a proven FBT to treat eating disorders in an outpatient family driven setting. The goal of the implementation framework was to achieve high fidelity to the proven FBT	17 individuals (nine therapists, four medical practitioners, four administration staff)	Therapists ± the other clinic staff
Henderson et al., 2017, Canada [15]	Mental Health and Substance Abuse	Youth serving network	Pre-implementation and implementation	Quantitative descriptive, post intervention qualitative feedback	To describe the process, the supports and barriers to implementation and lessons learnt from initiative in collaboration with community service providers	Pre-implementation: policymakers, local community leaders, organisational decision makers, direct service providers, administrative staff Implementation: The service providers	Healthcare providers in community youth services
Kingsley, 2020, USA [21]	Chronic pain	Paediatric, academic medical facility serving as a regional SCD center in the Midwest	Post-implementation	Quantitative non-randomized: Cohort study	Evidence based screening tool to increase multidisciplinary pain referrals for youth with SCD at risk for chronic pain	111 youth ages 2–21 years	An academic medical facility—MDT treating sickle cell
Nadeem et al., 2018, USA [25]	Mental health	School-based mental health clinics identified by New York City School–based Mental Health (NYC SBMH) Committee	Implementation monitoring and post implementation	Mixed Methods	1. Characterise the implementation activities and processes that occur within mental health clinics participating in a large scale school mental health training effort 2. Determine which processes relate to initial implementation outcomes 3. Utilise qualitative data to provide insights into the dynamic implementation processes that may underlie clinics' implementation behaviours as measured by the SIC	26 NYC school based mental health clinic sites which provide co-located school based mental health services	Therapists/social workers/psychiatrist (essentially all clinicians) working in school based mental health clinics
Radovic, 2019, USA [16]	Mental health	Two paediatric community practices	Pre-implementation	Mixed methods study—survey (quantitative) and focus groups	Develop and investigate potential implementation strategies for the introduction of Supporting Our Valued Adolescents (SOVA) web-based technology in 2 primary care settings with the goal of translating to more effective implementation in the future	14 PCP	PCPs in community practices who see/treat adolescents with depression and/or anxiety
Shafran et al., 2020, UK [27]	Mental health, epilepsy	Child health epilepsy services	Pre-implementation; implementation; post-implementation	Qualitative	To optimise MATCH-ADTC for use in children and young people with mental health needs in the context of epilepsy within routine epilepsy services, using implementation science methods	6 focus groups (FGs) of children and young people with epilepsy who had received treatment for epilepsy and 10 parents/carers 6 FGs of health professionals working in epilepsy services PDSA cycles with 12 patients receiving the version of the MATCH-ADTC intervention 8 parents participated in the qualitative interviews	Parents and children and young people with epilepsy Health clinicians
Stanhope et al., 2018, USA [17]	Treatment of substance use disorders	27 community mental health organizations (CMHOs) in 6 states	Post implementation, implementation monitoring	Mixed methods	1) Describe the implementation of SBIRT within CMHOs; and 2) understand the self-reported barriers to implementing SBIRT and when these barriers occurred in the implementation process	2873 adolescents screened, 55.1% female, average age 16.6 years (SD = 1.61).15–22	Staff of the community mental health services
Snider, 2016, Canada [23]	Violence/mental health	Community -Winnipeg's Health Science Centre	Pre-implementation	Qualitative	To describe how a group of community partners and medical professionals used an iKT approach to develop and EDVIP for youth injured by violence in Winnipeg's Health Sciences Centre and a research plan to evaluate it	The research team itself: Community partners: Youth workers, youth with lived experiences of violence (both as victims & perpetrators), Aboriginal Elders, executive directors of youth violence programs. Emergency & trauma doctors, nurses & social workers	Will be ED workers and community groups, possibly police
Westerlund, 2020, Sweden [18]	Mental Health	Children and Adolescent psychiatrist clinics	Post-implementation	Qualitative	1. Explore what extent the DA guidelines were known and adhered to by health professionals 2. Investigate factors influencing implementation of the guidelines	18 individuals from 3 separate child and adolescent psychiatry clinics (6 physicians, 6 social workers, 6 psychologists)	Clinicians—physicians, psychologists, social workers

^*^ EBT Evidence Based Training

^*^FBT Family-Based Therapy

^*^IS Implementation Science

^*^LC Learning Collaborative

^*^MDT Multidisciplinary Team

^*^OT Occupational Therapist

^*^PCP Primary care provider

^*^PT Physiotherapist

^*^SBIRT Screening, Brief Intervention and Referral to Treatment

^*^SCD Sickle Cell Disease

^*^SIC Stages of Implementation Completion

^*^TF-CBT Trauma-Focused Cognitive Behavioural Therapy

All the qualitative papers, two of the three quantitative, and four of the six mixed methods papers adequately addressed every question of the MMAT tool. Where paper quality was marked down, this was largely due to non-generalisable methods, issues of bias and incomplete reportage.

### Question 1: What implementation science theories, models and frameworks have been applied in support of service development, innovation or sustainability in adolescent healthcare?

Nine different implementation science theories, models or frameworks were applied in the adolescent healthcare research papers included in this review (Table 2), with eight of the twelve studies using a single theory, model or framework. The CFIR was the most popular, used alone in four studies [15–18], in combination with the Active Implementation Framework (AIF) and the Implementation Outcomes Taxonomy (IO) in two papers relating to the same study [19, 20], and in one study in combination with the PDSA framework [21]. Two studies used the Knowledge to Action (KTA) model alone [22, 23]. The Exploration, Preparation, Implementation, Sustainment (EPIS) framework and the Stages of Implementation Completion (SIC) framework were used alone in one study each [24, 25] and the EPIS was used with the National Centre for Child Traumatic Stress Learning Collaborative (NCCTS LC) model in another study [26]. One study used a combination of Plan, Do, Study, Act (PDSA) and the Normalization Process Theory (NPT) [27]. Of these cited approaches, KTA, EPIS, SIC, NCCTS LC and PDSA are categorised as ‘process’ (‘how to’) models, the AIF and the CFIR as ‘determinant’ (or explanatory) whilst the IO addresses evaluation and the NPT is a recent implementation theory [7].

**Table 2:** Use of theories, models and frameworks within each study

Study	IS theory/ model/ framework named as applied in the study	TMF used in planning and preparation activities?	TMF used in delivery of implementation processes?	TMF used in monitoring the implementation processes?	TMF used in evaluation of implementation process?	TMF used to plan/ enable sustainability?
Amaya-Jackson et al., 2018 [26]	National Center for Child Traumatic Stress (NCCTS) Learning Collaborative Model on the Adoption and Implementation of EBTs	Yes	Yes	Yes	Yes	Yes
Anaby et al., 2015 [22]	Knowledge to Translation	Yes	Yes	Yes	Yes	Not stated
	Participatory Action	'Principles used'	Not stated	Not stated	Not stated	Not stated
Beidas et al., 2016 [24]	EPIS	Yes	Yes	Yes	Yes	Out of scope for paper
Couturier et al., 2018 [19]	AIF	Reported elsewhere	Yes	Yes	Yes	Not stated
	CFIR	Not mentioned	Not mentioned	Not mentioned	Not mentioned	Not mentioned
	IO	Not mentioned	Not mentioned	Not mentioned	Not mentioned	Not mentioned
Couturier et al., 2021 [20]	AIF	Yes	Yes	Yes	No	Out of scope for paper
	CFIR	Yes	Yes	No	Yes	Out of scope for paper
	IO	No	No	No	Yes	Out of scope for paper
Henderson et al., 2017 [15]	CFIR	Yes	Yes	Yes	Yes	Not stated
Kingsley, 2020 [21]	PDSA	'Stated but not explained'	Not stated	Not stated	Not stated	Out of scope for paper
	CFIR	'Stated but not explained'	No	No	No	Out of scope for paper
Nadeem et al., 2018 [25]	SIC	Yes	Yes	Yes	Yes	Stated but detail unclear
Radovic, 2019 [16]	CFIR	Yes	Not stated	No	Yes	Not stated
Shafran et al., 2020 [27]	NPT	Yes	Yes	Yes	Yes	Not stated
	PDSA	Yes	Yes	Yes	Yes	Not stated
Stanhope et al., 2018 [17]	CFIR	No	No	Yes	Yes	Not stated
Snider, 2016 [23]	Knowledge to Action	Yes	Yes	Yes	Yes	Out of scope for paper
Westerlund, 2020 [18]	CFIR	No	No	No	Yes	Out of scope for paper

^*^AIF Active Implementation Framework

^*^CFIR Consolidated Framework for Implementation Research

^*^EPIS Exploration, Preparation, Implementation, Sustainment

^*^ IO Implementation Outcomes Taxonomy

^*^NPT Normalisation Process Theory

^*^PDSA Plan, Do, Study, Act

^*^SBIRT Screening, Brief Intervention and Referral to Treatment

^*^SCD Sickle Cell Disease

^*^SIC Stages of Implementation Completion

^*^TF-CBT Trauma-Focused Cognitive Behavioural Therapy

These theories, models and frameworks were applied at various points within studies’ trajectories: in planning and preparation and in delivery of the intervention implementation strategy and processes; in monitoring and evaluation of these implementation strategies; to plan for and enable sustainability of change (Table 2). One study only applied a framework (the CFIR) during evaluation, using it as a post-hoc framework for analysis [18]; another study claimed both the CFIR and PDSA were used for planning purposes but did not explain how [21]. One study used the CFIR for both planning and evaluation [16]. All other studies applied one or more theory, model or framework at multiple points through the study trajectory, often providing very detailed accounts of project development, delivery and evaluation where these approaches were integral and essential elements. Their use in relation to sustainability of change, however, was only mentioned in one study [25], although for at least five papers, this could be considered outside the scope of the specific publication.

### Question 2: What elements of these frameworks have been identified as influential in promoting the implementation and sustainability of service intervention?

Findings in response to Question 2 are mapped in Table 3, synthesised in reference to the CIFR domains of 1) Intervention characteristics, 2) Outer setting, 3) Inner setting, 4) Individual characteristics, and 5) Process [5].

**Table 3:** Significant supports and barriers to implementation identified in included papers and aligned to CFIR domains and factors

CFIR Domain	CFIR Domain Factor	Facilitators	*n*	Barriers	*n*
**Intervention characteristics**	**Intervention Source**	(Kingsley 2020) [21]	1	(Westerlund et al. 2020) [18]	1
	**Evidence Strength and Quality**	(Amaya-Jackson et al. 2018; Nadeem et al. 2018; Kingsley 2020) [26,25,21]	3	(Kingsley 2020; Westerlund et al. 2020) [21,18]	2
	**Relative Advantage**	(Anaby et al. 2015; Radovic et al. 2019; Westerlund et al. 2020; Couturier et al. 2021) [22,16,18,20]	4	(Nadeem et al. 2018) [25]	1
	**Adaptability**	(Stanhope et al. 2018; Radovic et al. 2019; Kingsley 2020; Shafran et al. 2020) [17,16,21,27]	4	(Beidas et al. 2016; Couturier et al. 2018; Stanhope et al. 2018; Radovic et al. 2019; Westerlund et al. 2020; Couturier et al. 2021) [24,19,17,16,18,20]	6
	**Trialability**	-	0	-	0
	**Complexity**	(Nadeem et al. 2018; Kingsley 2020) [25,21]	2	(Anaby et al. 2015; Beidas et al. 2016; Henderson et al. 2017; Couturier et al. 2018; Nadeem et al. 2018; Radovic et al. 2019; Kingsley 2020; Shafran et al. 2020) [22,24,15,20,25,16,21,27]	8
	**Design Quality and Packaging**	(Anaby et al. 2015: Radovic et al. 2019; Kingsley 2020; Couturier et al. 2021) [22,16,21,20]	4	(Radovic et al. 2019) [16]	1
	**Cost**	(Amaya-Jackson et al. 2018) [26]	1	(Amaya-Jackson et al. 2018) [26]	1
**Outer setting**	**Patient Needs and Resources**	(Nadeem et al. 2018; Radovic et al. 2019; Shafran et al. 2020) [25,16,27]	3	(Beidas et al. 2016; Amaya-Jackson et al. 2018; Couturier et al. 2018; Nadeem et al. 2018; Stanhope et al. 2018; Kingsley 2020; Shafran et al. 2020; Westerlund et al. 2020) [24,26,19,25,17,21,27,18]	8
	**Cosmopolitanism**	-	0	-	0
	**Peer Pressure**	-	0	-	0
	**External Policies and Incentives**	-	0	(Beidas et al. 2016; Stanhope et al. 2018) [24,17]	2
	**Structural Characteristics**	(Kingsley 2020) [21]	1	(Beidas et al. 2016; Couturier et al. 2018; Nadeem et al. 2018; Stanhope et al. 2018) [24,19,25,17]	4
	**Networks and Communications**	(Henderson et al. 2017; Amaya-Jackson et al. 2018; Couturier et al. 2018; Radovic et al. 2019) [15,26,19,16]	4	(Radovic et al. 2019; Westerlund et al. 2020) [16,18]	2
	**Culture**	(Anaby et al., 2015) [22]	1	(Anaby et al. 2015; Kingsley 2020; Westerlund et al. 2020) [22,21,18]	3
	**Implementation Climate**	(Anaby et al. 2015; Westerlund et al. 2020) [22,18]	2	(Beidas et al. 2016; Nadeem et al. 2018; Stanhope et al. 2018; Westerlund et al. 2020) [24,25,17,18]	4
	**Tension for Change**	(Radovic et al. 2019; Westerlund et al. 2020) [16,18]	2	(Westerlund et al. 2020) [18]	1
	**Compatibility**	(Radovic et al. 2019; Kingsley 2020) [16,21]	2	(Stanhope et al. 2018; Westerlund et al. 2020) [17,18]	2
	**Relative Priority**	(Kingsley 2020) [21]	1	(Anaby et al. 2015) [22]	1
	**Organizational Incentives and Rewards**	(Beidas et al. 2016) [24]	1	(Beidas et al. 2016) [24]	1
	**Goals and Feedback**	-	0	-	0
	**Learning Climate**	(Radovic et al. 2019) [16]	1	-	0
	**Readiness for Implementation**	(Radovic et al. 2019) [16,19]	1	-	0
	**Leadership Engagement**	(Beidas et al. 2016; Nadeem et al. 2018) [24,25]	2	(Nadeem et al. 2018) [25]	1
	**Available Resources**	(Beidas et al. 2016; Henderson et al. 2017; Amaya-Jackson et al. 2018; Nadeem et al. 2018; Stanhope et al. 2018; Couturier et al. 2021; Kingsley 2020) [24,15,26,25,17,20,21]	7	(Anaby et al. 2015; Beidas et al. 2016; Henderson et al. 2017; Amaya-Jackson et al. 2018; Couturier et al. 2018; Nadeem et al. 2018; Stanhope et al. 2018; Radovic et al. 2019; Couturier et al. 2021; Kingsley 2020; Shafran et al. 2020; Westerlund et al. 2020) [22,24,15,26,19,25,17,16,20,21,27,18]	12
**Inner setting**	**Access to Knowledge and Information**	-	0	-	0
	**Knowledge and Beliefs about the Intervention**	(Anaby et al. 2015; Radovic et al. 2019) [22,16]	2	(Nadeem et al. 2018; Kingsley 2020; Westerlund et al. 2020) [25,21,18]	3
	**Self-efficacy**	(Radovic et al. 2019) [16]	1	-	0
	**Individual Stage of Change**	(Anaby et al. 2015) [22]	1	-	0
	**Individual Identification with Organization**	-	0	-	0
**Individual characteristics**	**Other Personal Attributes**	(Anaby et al. 2015) [22]	1	-	0
	**Planning**	(Beidas et al. 2016; Snider et al. 2016; Amaya-Jackson et al. 2018; Kingsley 2020) [24,23,26,21]	4	(Beidas et al. 2016) [24]	1
	**Engaging**	(Anaby et al., 2014; Beidas et al. 2016; Snider et al. 2016; Henderson et al. 2017; Amaya-Jackson et al., 2018; Couturier et al. 2018; Nadeem et al. 2018; Stanhope et al. 2018; Couturier et al. 2021; Radovic et al. 2019; Kingsley 2020; Shafran et al. 2020) [22,24,23,15,26,19,25,17,20,16,21,27]	12	(Nadeem et al. 2018; Westerlund et al., 2020) [25,18]	2
	**Opinion Leaders**	(Nadeem et al. 2018; Kingsley 2020) [25,21]	2	-	0
	**Formally Appointed Internal Implementation Leaders**	(Nadeem et al. 2018; Stanhope et al. 2018; Couturier et al. 2021) [25,17,20]	3	-	0
	**Champions**	(Nadeem et al. 2018) [25]	1	-	0
	**External Change Agents**	-	0	-	0
	**Executing**	(Amaya-Jackson et al. 2018) [26]	1	(Kingsley 2020) [21]	1
**Process**	**Reflecting and Evaluating**	(Anaby et al. 2015; Couturier et al. 2021) [22,20]	2	(Couturier et al. 2021) [20]	1

### Intervention characteristics

Eleven papers [15–19, 21, 22, 24–27] acknowledged the contribution of intervention characteristics as barriers or facilitators to implementation. Two studies cited the *intervention source* [18, 21], noting that where key stakeholders and end-users were involved in development, this facilitated uptake. By contrast, where externally developed (mental health) guidelines showed limited implementation success, this was attributed to developers’ oversimplified understanding of the population and issues [18].

Stakeholder perceptions regarding *evidence strength and quality* were important [18, 21, 25, 26], whether based on clear theoretical or research grounding [25] or expert opinion [21]. Scepticism from health professionals regarding supporting evidence was a barrier to implementation [18]. Demonstrated evidence of program effectiveness was important for securing continued funding for one intervention [26].

The *relative advantage* of the intervention compared to available alternatives was credited as influential [16, 18, 20, 22, 25]. Uptake was enhanced where health professionals believed that implementation could improve the quality of care and services, reduce practice variations, and promote job satisfaction and professionalism [16, 18, 20, 22]. Perceived relative advantage was high where there was a match between the intervention and recognised patient, clinician and/or service needs [16, 18, 20, 22], and where there were no comparable interventions [16]. Barriers related to relative disadvantage arose from difficulties obtaining buy-in, service billing issues, and time [25].

Recommendations and actions to improve the *adaptability* of interventions included modifying and streamlining processes to suit work practices [21], developing workarounds for technical issues [17], personalising interventions for the individual and context [27], and adapting how interventions were introduced to healthcare professionals [16]. Barriers to adaptability [16–20, 24] related to difficulties in tailoring interventions to suit populations, contexts, and workflows [17–19, 24], in training staff across broad services [20], in integrating technologies (e.g. with electronic medical records, mobile applications) [16, 17] and confidentiality concerns [17].

The *perceived complexity of the intervention* was important [15, 16, 19, 21, 22, 24, 25, 27]. Implementation was easier where interventions were straightforward and easy to understand, and could be applied without much additional effort or impact on current workflows [21, 25]. Time and resource constraints hindered planning, training, implementation and evaluation [15, 19, 22, 24, 25], as did additional and/or unnecessary processes and workload requirements [15, 21, 22, 24], poor intervention fit with daily routines and competing priorities [16, 22], lack of role clarity and role overlaps [19], and the general invasiveness of the intervention [27]. *Cost* was also a factor [26].

*Intervention design, quality and presentation* were typically positive attributes [16, 20–22]. Clinicians praised translation workshops for multi-disciplinary attendance, and the relevance and usefulness of content including knowledge translation processes [22]. The involvement of both medical practitioners and administrators in training, the use of role-play, the consistent review of treatment sessions, and frequent and immediate feedback were appreciated [20].

### Outer setting

Nine studies cited influential factors from the outer setting [16–19, 21, 24–27]. *Patient needs and resources* were most frequently cited [16–19, 21, 24–27]. Patients needed clinicians to be trained in evidence-based practice and relevant specialist services [25] and for technological approaches to service provision such as telehealth to be available [27]. Barriers arose from the complex and diverse needs of young people including their comorbidities, unstable home lives, trauma and other risk factors [17–19, 24, 26]. Patient-related difficulties arose in identifying and locating young people who met intervention criteria [17, 21, 24], from patient (un)readiness for treatment [17] and disruptive behaviours [24] and from dropout due to unstable home life or geographical relocation [26]. Services were challenged by the stigma of mental health [16] and the difficulties of delivering services other than in person (such as via telehealth or telephone) [27].

*External policies and incentives* that presented barriers included the impact of government policies and regulations on billing practices and burdens placed on services due to licencing and regulatory demands [17]. Closure of services also affected implementation [24].

### Inner setting

Ten papers cited factors related to the inner setting [15–19, 21, 22, 24–26]. Supportive *structural characteristics* included established relationships between stakeholders and infrastructure such as staff shared across departments and allotted clinic time for the intervention [21]. Structural hindrances included staffing turnover and scheduling, large caseloads and lengthy clinic waitlists, difficult intake and billing processes [17, 19, 25].

Existing *networks and communications* could be capitalised on [15, 16, 19, 26], for example, by supporting role clarity [19] and enabling communication between primary care and nurse coordinators to monitor patient progress [16]. Formal and informal collaboration across teams, knowledge sharing between clinicians or health agencies on how to address implementation barriers [15, 26], joint educational opportunities and use of common tools [15] were cited. Relationship building was important [15, 16, 19, 26], and strong positive relationships between stakeholders were a success factor [15]. Conversely, lack of formal communication systems between stakeholders [18] and slow responses [16, 18] were barriers and accrued negative consequences for the care of young people [18].

*Organisational cultures and values* both hindered and facilitated knowledge translation [18, 21, 22]. Cultures that valued continuing education and learning and sought to link research to practice supported implementation [22], whereas a culture of autonomy amongst clinicians could hinder guideline implementation [18] and mean that evidence-informed referrals were perceived as unnecessary [21].

The *implementation climate* was described as important [18, 22, 24, 25], and comprised *tension for change, compatibility, relative priority, organisational incentives and rewards, goals and feedback* and *the learning climate* (Table 3). The commitment and involvement of leaders throughout the course of implementation [18], stakeholder buy-in and an organisational mandate [22] were characteristics of a positive climate. A negative climate was seen in lack of agreement on the prioritisation of activities [18], where practical issues deterred leadership support [24] and where there was a general perception that an intervention was too difficult [18, 24, 25].

*Tension for change* was in evidence where change was perceived to increase the quality of care, reduce practice variations and improve work settings [18] or could be presented as responding to negative media [16]. Tension for change was low when the perceived need was also low [18]. The organisational *learning climate* was cited as a facilitator when primary care physicians were seen to actively seek education opportunities [16].

Where a project aligned with organisational goals without overlapping other activities, the perceived *compatibility* of the intervention facilitated implementation [16, 21]. However, a poor match to health professional and patient populations [18] and competing systems and changes within an organisation [17] had a negative impact on facilitating change. The *relative priority* attributed to an intervention depended on the support of key stakeholders [21, 22] and competing priorities [17].

Two studies recorded *organizational incentives and rewards* as influential. In one, stipends and acknowledgement rewarded individuals who worked as brokers between the implementation team and the front-line implementers. By contrast, not allowing therapists to bill at an enhanced rate unless implementing an intervention with complete fidelity was a clear barrier [24]. In the other study the lack of incentives, goals and feedback systems deterred implementation [18].

*Readiness for implementation* comprised *leadership engagement, available resources* and *access to knowledge and information. Readiness for implementation* could be indicated variously: by clinicians’ personal values about the topic and of continuing learning and education, by clinicians’ curiosity, and desire to link research to practice or validate clinical wisdom, and by peer endorsements [22]. *Leadership engagement* approaches that involved quick decision-making with limited stakeholder consultation and sub-optimal communication of changes were ineffectual [25], whereas successful implementation featured more thoughtful and engaged decision-making processes involving numerous stakeholders at multiple levels. Successful initiatives involved executive leadership buy-in [24] and engaged program administrators, who advocated for change upwards through the approval chain, and emphasised the fit of the intervention with the mission of the organisation [25]. A broker between the implementation team and the front-line implementers was also helpful [24]. Studies cited *available resources* as critical for implementation [15–22, 24–27], with resource barriers and facilitators deriving from staffing, workloads, training, physical space, funding and time.

### Individual characteristics

Five studies cited the contribution of *individual characteristics* as either barriers or facilitators to implementation [16, 18, 21, 22, 25], with *stakeholder knowledge and beliefs about the intervention* the most commonly reported factor [16, 18, 21, 22, 25]. One study revealed that lack of knowledge could facilitate implementation by motivating clinicians to learn [22], whilst others found negative beliefs about the need for and/or utility of interventions was a barrier to implementation [18, 21, 25]. S*elf-efficacy* was mentioned in three studies [16, 20, 26], with two citing null results (not reported in Table 3) [20, 26] and one indicating this factor facilitated implementation [16]. *The individual stage of change* was mentioned in one study that linked this to positive intentions for change [22]. *Other personal attributes* facilitating implementation included personal values regarding learning and education, and curiosity [22], but neither prior knowledge and experience [26] nor attitudes and readiness [20] affected implementation.

### The implementation process

*Planning* was critical for implementation [21, 23, 24, 26] and entailed starting early, screening for and identifying intervention recipients and key stakeholders, undertaking tailored consultations, training staff and trialling tools [21, 24, 26]. Project team meetings were opportunities to share information, build trust and discuss issues [23, 24]. Ensuring the right team members was important: for example, appointing a support worker for an intervention for youth injured by violence with “lived experience” or significant relevant work experience [23].

Implementation was supported by the *engagement* of a range of facilitatory roles, achieved via multiple diverse strategies. *Engaging* was a critical aspect of implementation, raised by all 13 papers variously in relation to *opinion leaders, formally appointed internal implementation leaders, champions and external change agents* [15–27]. Engagement processes were primarily discussed in positive terms [15–17, 19–27] although two studies highlighted the negative impacts of limited stakeholder engagement [18, 25].

For all these roles, creating opportunities to build relationships and learn together were key implementation strategies. One study found that inter-state learning communities and mutual support assisted engagement and implementation [17]. Engaging support from respected clinicians and managers at various levels also facilitated implementation [21]. Thoughtful and involved decision-making processes engaged senior executives, ‘selling’ them on the project so they advocated up the chain for approval [25].

Having medical practitioners and administrators present at training workshops provided opportunities for relationship-building [20]. Inter-sectoral and joint meetings acted as educational and capacity-building events that promoted information sharing, goal setting and opportunities for stakeholders to connect [15]. Positive relationships between front-line staff and implementation teams were sustained by project leads participating in site visits and webinars [15]. The continued engagement of key stakeholders who were influential opinion leaders was facilitated via timely feedback including communication of progress [21]. A lack of engagement was highlighted as a barrier in one study where decision-making processes were abrupt with minimal stakeholder involvement [25].

*Execution* of implementation was considered in several papers. Barriers included unplanned staff absences and leave which had ramifications for workflows [21]. Facilitators included funding that provided for extended training and time for trainers to spend with trainees [26]. *Reflecting and evaluating* was illustrated in studies where implementation teams focused on unanticipated negative outcomes and how these could be addressed [20, 22]. One study built in reflection and recap processes following the intervention [22], while another considered suggestions from participants [20].

### Question 3: To what extent and in what capacity have the contribution of adolescent consumer perspectives on evidence implementation been identified or reported in the development and application of implementation frameworks? At what time points were adolescent perspectives considered?

Review inclusion criteria specified that either adolescents or providers of healthcare services designed for adolescents should be targeted in included studies. In all included papers young people or adolescents and children were the recipients of the clinical interventions, but the target of reported implementation strategies was most often exclusively the clinicians and staff delivering it [15, 16, 18–20, 22, 24, 25]. For example, in Couturier and colleagues’ papers [19, 20], the intervention recipients were adolescents aged 12–18, but therapists’ fidelity to the family based therapy protocol was the study outcome. Similarly, Stanhope and colleagues implemented their Screening, Brief Intervention and Referral to Treatment intervention in young people aged 15–22, but clinicians rather than adolescents were asked to evaluate it. Three papers reported data from both clinicians/ staff and adolescent consumers [23, 26, 27] and two papers used adolescents’ routinely collected data or generated new process records [17, 21].

Across the 13 included papers there was little or no inclusion of adolescents or youth in the development or review of any health service intervention or implementation strategy. Only three studies mentioned adolescent input, which occurred primarily in the pre-implementation stage of the studies, or took place while implementation was underway. Shafran and colleagues held focus groups to discuss issues related to engagement and the delivery of their intervention, both prior and during the intervention, which included five young people [27]. Radovic and colleagues consulted a youth research advisory board for feedback on their proposed implementation strategy. The adolescents’ feedback differed on some points from that of the clinicians, demonstrating the importance of including the perspectives of young people [16]. Finally, Snider and colleagues spoke to ‘youth with lived experience with violence’ while developing their violence intervention program [23]. These young people offered insight into their experiences in the Emergency Department, describing how vulnerable they felt during this hospital presentation. Clinicians suggested that this might indicate a ‘teachable moment’ and this led to the decision to implement the violence intervention program in Emergency Departments, rather than in community settings.

In summary, while adolescents were the eventual recipients of all interventions, their input or feedback was rarely sought across the included studies. When included, there was evidence that the voice of young people was able to guide how interventions or implementation could be improved.

## Discussion

An important finding of this review is the international sparsity of work in this field, with only 13 papers from 12 studies included, 10 from North America. Whilst this review did not aim to present a comprehensive view of all implementation activities across youth health services, the small number of theoretically underpinned studies makes clear the under-developed nature of the topic. Eight of these twelve studies described application of implementation science theory, models and frameworks within at least three of the major stages of implementation work: in project planning, delivery, monitoring and evaluation, with sustainability barely mentioned. Overall, these were credible accounts of theory integral to the processes of practice innovation and change.

The extensively referenced CFIR (4,251 citations at October 2021) was chosen to support this structured analysis of implementation supports and barriers because its menu of constructs captures the complexity and multi-level nature of implementation [28]. The CFIR has been used as a theoretical framework to generate context-specific logic models (i.e. targeted and tailored), and as a pragmatic guide to methodically assess and evaluate facilitators and barriers in developing and delivering innovations (see https://cfirguide.org/). Whilst predominantly applied in adult studies, CFIR has also been used for paediatric services and adapted for use in school settings [29], indicating relevance across age groups. However, no age-appropriate adaptation for adolescence was found.

Unique challenges and barriers deriving from the characteristics of adolescent populations were repeatedly flagged, and this is an important consideration for service developers [16, 18, 19, 21, 26]. Most health systems are binary, designated either as paediatric or adult where age defines access, but neither system is ideally positioned to support the morbidities of adolescents. These morbidities are those associated with health risk behaviours, emerging non-communicable chronic disease and mental health, as broadly reflected in this review. Too often adolescents are stigmatised and stereotyped in the general community as risk takers, irresponsible and generally difficult to engage. Unfortunately, this stigma is also present in health systems and services, as well as in research, where adolescents are often considered too challenging to work with [30]. The majority of adolescent healthcare, other than for non-communicable chronic disease, takes place in primary care and other community settings, Emergency Departments or in specialised services for high risk or marginalised youth, and for mental health, also reflected in the studies in this review. Many services for adolescents are under-resourced from the beginning and implementation studies are likely viewed as an additional burden, rather than a way to improve patient outcomes.

Many of the studies reported on their preparatory stages, most often from the provider perspective; in these situations, no comment can be made on the value of the work to the end user. This is reflective of the wider situation, in which advocacy for the specific health needs of adolescents is commonly missing from policy and practice, and adolescents are often not allowed a voice [31]. In the few reviewed studies that included adolescents in the implementation process, their input was episodic and not consistent throughout the process; it could be considered tokenistic or, at best, only briefly reported. There is increasing acknowledgement of the importance of the consumer voice in healthcare and this is perhaps particularly the case for implementation studies. Without this, even an intervention perfectly implemented as planned may not achieve the desired outcomes.

In summary, adolescents and youth too-often miss out or are overlooked and are the losers in health systems, and this review makes clear that this includes implementation work. Together, the above findings indicate that implementation managers should consider a number of factors when planning, implementing, scaling-up and evaluating health service interventions for adolescents. The most commonly cited intervention characteristics that facilitated overall implementation were the relative advantage, adaptability and design, quality and packaging of interventions. This suggests that implementation strategies for youth health service interventions must consider how any proposed intervention will work in the ‘real world’. Knowledge translators must know not just why a proposed intervention is superior to other options, how it can be adapted to the specific context and how this can be ‘sold’ to individuals charged with implementation but also what is required to meet the needs and preferences of the adolescent consumer. The most commonly cited barriers to implementation were the complexity and adaptability of the interventions. Whilst this may reflect a bias of researchers for whom the design elements of an intervention may be a familiar focus, this may also flag the challenges of trying to adapt systems and processes to meet the needs of adolescents without or with minimal youth input to inform this.

### Limitations and strengths of the review

In the realist world of service and practice innovation, implementation research juggles the methodology issues that attend pragmatic trials, mixed methods designs and complex interventions, compounded by the frequent need to measure implementation outcomes for which no standardised instruments exist [32]. Included studies all experienced these challenges and their variable successes at meeting them are reflected in their quality scores.

This review aimed to identify what elements of established implementation science theories, models and frameworks have been reported as enablers or barriers in implementing interventions in adolescent health services. It did not seek to map all factors reported as influential, as, in the absence of linkage to this epistemology, the generalisability of findings would be impossible to gauge. This may mean that by applying a framework developed from adult evidence, adolescent-specific factors may have been missed. Future review of studies carried out from pragmatic rather than theoretical foundations may reveal new knowledge. However, the comprehensive nature of the CFIR guided its choice to structure analysis of the reported enablers and barriers, despite this framework’s predominantly adult provenance. An advantage was that this revealed the substantial degree of common ground in the implementation field for factors influential for adolescent as well as adult health service implementation. The process of classifying barriers and facilitators to implementation using the CFIR was a largely subjective process, mitigated by using two reviewers for this task. The count of CFIR domain factors cannot be used to ‘weight’ the importance of specific CFIR implementation influences within the adolescent health space and it should be borne in mind that just because a factor is not mentioned does not mean it is unimportant. A number of studies did not mention anticipated influences on implementation but this could be because they entailed assumed knowledge (thought to be obvious) or were not considered worth mentioning by the researcher. This does not necessarily mean that these processes did not occur. Indeed, some aspects of implementation (such as intervention cost) may be so central that they are not discussed because the intervention would never be supported to even pre-implementation stage if it did not meet this threshold.

## Conclusion

The facilitators and barriers flagged by this review are broadly generic; inadequate resourcing, lack of policy direction and leadership, lack of interest in improving a system for ultimate end users are consistent implementation themes which need to be resolved when implementing change in adolescent healthcare. The lack of adolescent consumer input, and limited recognition and/or inclusion of the specific developmental needs of adolescence, such as neurocognitive development, psychosocial resources and evolving autonomy, represent a start point in addressing the stark research deficit revealed by this review.

## Methods

All methods were performed in accordance with the relevant guidelines and regulations.

## Supplementary Information

**Additional file 1.** 

**Additional file 2.** 

**Additional file 3.**

## Data Availability

Publicly available data only were used in this review and all are referenced appropriately in the manuscript.

## References

[1] Morris ZS, Wooding S, Grant J (2011). The answer is 17 years, what is the question: understanding time lags in translational research. J R Soc Med.

[2] Grimshaw JM, Eccles MP, Lavis JN, Hill SJ, Squires JE (2012). Knowledge translation of research findings. Implement Sci.

[3] Runciman WB, Hunt TD, Hannaford NA, Hibbert PD, Westbrook JI, Coiera EW (2012). CareTrack: assessing the appropriateness of health care delivery in Australia. Med J Aust.

[4] Greenhalgh T, Robert G, Macfarlane F, Bate P, Kyriakidou O (2004). Diffusion of innovations in service organizations: systematic review and recommendations. Milbank Q.

[5] Damschroder LJ, Aron DC, Keith RE, Kirsh SR, Alexander JA, Lowery JC (2009). Fostering implementation of health services research findings into practice: a consolidated framework for advancing implementation science. Implement Sci.

[6] CFIR Research Team (2021) Articles & Highlights. *CFIR Guide.* Retrieved from https://cfirguide.org/articles-highlights/. Accessed on 1/10/21

[7] Nilsen P. Making sense of implementation theories, models and frameworks. Implementation Science. 2015;10(53). 10.1186/s13012-015-0242-0 PMC4406164 25895742

[8] Agency for Clinical Innovation (2021) Accelerating Implementation Methodology. Retrieved from https://aci.health.nsw.gov.au/make-it-happen/centre-for-healthcare-redesign/accelerating-implementation-methodology-aim. Accessed on 1/10/21

[9] Whittemore R, Knafl K (2005). The integrative review: updated methodology. J Adv Nurs.

[10] Page MJ, McKenzie JE, Bossuyt PM, Boutron I, Hoffmann TC, Mulrow CD, The PRISMA (2020). statement: an updated guideline for reporting systematic reviews. BMJ.

[11] DiCenso A, Guyatt G, Ciliska D. Evidence-based nursing: A guide to clinical practice: Elsevier Health Sciences; 2005.

[12] Covidence systematic review software, Veritas Health Innovation, Melbourne, Australia. Available at www.covidence.org

[13] Hong Q, Pluye P, Fàbregues S, Bartlett G, Boardman F, Cargo M (2018). Mixed methods appraisal tool (MMAT) version 2018: user guide.

[14] Braun V, Clarke V (2006). Using thematic analysis in psychology. Qual Res Psychol.

[15] Henderson JL, Chaim G, Brownlie E (2017). Collaborating with community-based services to promote evidence-based practice: Process description of a national initiative to improve services for youth with mental health and substance use problems. Psychol Serv.

[16] Radovic A, Odenthal K, Flores AT, Miller E, Stein BD (2020). Prescribing technology to increase uptake of depression treatment in primary care: a Pre-implementation focus group study of SOVA (supporting our valued adolescents). J Clin Psychol Med Settings.

[17] Stanhope V, Manuel JI, Jessell L, Halliday TM (2018). Implementing SBIRT for adolescents within community mental health organizations: A mixed methods study. J Subst Abuse Treat.

[18] Westerlund A, Ivarsson A, Richter-Sundberg L (2021). Evidence-based practice in child and adolescent mental health services–The challenge of implementing national guidelines for treatment of depression and anxiety. Scand J Caring Sci.

[19] Couturier J, Kimber M, Barwick M, Woodford T, McVey G, Findlay S (2018). Themes arising during implementation consultation with teams applying family-based treatment: a qualitative study. J Eat Disord.

[20] Couturier J, Kimber M, Barwick M, Woodford T, Mcvey G, Findlay S (2021). Family-based treatment for children and adolescents with eating disorders: a mixed-methods evaluation of a blended evidence-based implementation approach. Translational Behavioral Medicine.

[21] Kingsley RA (2020). A Healthcare Improvement Initiative to Increase Multidisciplinary Pain Management Referrals for Youth with Sickle Cell Disease. Pain Management in Nursing.

[22] Anaby D, Korner-Bitensky N, Law M, Cormier I (2015). Focus on participation for children and youth with disabilities: Supporting therapy practice through a guided knowledge translation process. Br J Occup Ther.

[23] Snider C, Woodward H, Mordoch E, Chernomas W, Mahmood J, Wiebe F (2016). Development of an emergency department violence intervention program for youth: an integrated knowledge translation approach. Progress in Community Health Partnerships: Research, Education, and Action.

[24] Beidas RS, Adams DR, Kratz HE, Jackson K, Berkowitz S, Zinny A (2016). Lessons learned while building a trauma-informed public behavioral health system in the City of Philadelphia. Eval Program Plann.

[25] Nadeem E, Saldana L, Chapman J, Schaper H (2018). A mixed methods study of the stages of implementation for an evidence-based trauma intervention in schools. Behav Ther.

[26] Amaya-Jackson L, Hagele D, Sideris J, Potter D, Briggs EC, Keen L (2018). Pilot to policy: statewide dissemination and implementation of evidence-based treatment for traumatized youth. BMC Health Serv Res.

[27] Shafran R, Bennett S, Coughtrey A, Welch A, Walji F, Cross JH, et al. Optimising evidence-based psychological treatment for the mental health needs of children with epilepsy: principles and methods. Clinical Child and Family Psychology Review. 2020:1–12. 10.1007/s10567-019-00310-3 PMC7192863 31965422

[28] Shortell SM (2004). Increasing value: a research agenda for addressing the managerial and organizational challenges facing health care delivery in the United States. Medical Care Research and Review.

[29] Hudson KG, Lawton R, Hugh-Jones S (2020). Factors affecting the implementation of a whole school mindfulness program: a qualitative study using the consolidated framework for implementation research. BMC Health Serv Res.

[30] Noori O, Jackson M, Shetty A, Medlow S, Steinbeck K (2019). Transition care and the emergency department. Emerg Med Australas.

[31] Collin P, McCormack J. Young people and democracy: a review. Whitlam Institute; 2020.

[32] Newhouse R, Bobay K, Dykes PC, Stevens KR, Titler M (2013). Methodology issues in implementation science. Med Care.

