# Supplementary material for: Implementation science in adolescent healthcare research: an integrative review
Source: BMC Health Serv Res. 2022 May 3;22:598. doi: 10.1186/s12913-022-07941-3 (PMC9066920; doi:10.1186/s12913-022-07941-3)
Supplement: Supplementary file 1 — Additional file 1. [file 12913_2022_7941_MOESM1_ESM.docx]

## **SUPPLEMENTARY FILE 1: Search Strategies**

**Database: Embase Classic <1947 to 1973>, Embase <1974 to 2019 July 01>**

Search Strategy:

--------------------------------------------------------------------------------

1 youth*.tw. (83988)

2 adolescen*.tw. (339862)

3 young person*.tw. (5588)

4 1 or 2 or 3 (394782)

5 (research and practice and (translation or translating or translate*)).tw. (8930)

6 (knowledge adj1 (tranfer* or translation* or exchange* or disseminat* or diffusion* or innovation* or implement*)).tw. (5042)

7 implementation science*.tw. (1290)

8 implementation science/ (378)

9 (evidence based adj4 (diffusion* or innovation* or implement* or practice*)).tw. (24666)

10 5 or 6 or 7 or 8 or 9 (37770)

11 4 and 10 (1406)

12 limit 11 to (human and english language and yr="2009 -Current") (1142)

13 limit 12 to adolescent <13 to 17 years> (690)

**Database: Ovid MEDLINE(R) ALL <1946 to June 27, 2019>**

Search Strategy:

--------------------------------------------------------------------------------

1 youth*.tw. (67413)

2 adolescen*.tw. (256419)

3 young person*.tw. (3292)

4 1 or 2 or 3 (299658)

5 (research and practice and (translation or translating or translate*)).tw. (6665)

6 (knowledge adj1 (tranfer* or translation* or exchange* or disseminat* or diffusion* or innovation* or implement*)).tw. (3684)

7 implementation science*.tw. (1159)

8 implementation science/ (130)

9 (evidence based adj4 (diffusion* or innovation* or implement* or practice*)).tw. (19511)

10 5 or 6 or 7 or 8 or 9 (29202)

11 4 and 10 (1096)

12 limit 11 to (english language and humans and yr="2009 -Current") (652)

13 limit 12 to "adolescent (13 to 18 years)" (529)

**Database: PsycINFO <1806 to June Week 4 2019>**

Search Strategy:

--------------------------------------------------------------------------------

1 youth*.tw. (95266)

2 adolescen*.tw. (246625)

3 young person*.tw. (2518)

4 1 or 2 or 3 (303165)

5 (research and practice and (translation or translating or translate*)).tw. (4275)

6 (knowledge adj1 (tranfer* or translation* or exchange* or disseminat* or diffusion* or innovation* or implement*)).tw. (2057)

7 implementation science*.tw. (691)

8 implementation science/ (0)

9 (evidence based adj4 (diffusion* or innovation* or implement* or practice*)).tw. (14883)

10 5 or 6 or 7 or 8 or 9 (20642)

11 4 and 10 (2205)

12 limit 11 to (english language and humans and yr="2009 -Current") [Limit not valid in PsycINFO; records were retained] (1643)

13 limit 12 to "adolescent (13 to 18 years)" [Limit not valid in PsycINFO; records were retained] (1643)

14 limit 13 to (human and english language and yr="2009 -Current") (1625)

15 limit 14 to 200 adolescence <age 13 to 17 yrs> (720)
