# Supplementary material for: Implementation science in adolescent healthcare research: an integrative review
Source: BMC Health Serv Res. 2022 May 3;22:598. doi: 10.1186/s12913-022-07941-3 (PMC9066920; doi:10.1186/s12913-022-07941-3)
Supplement: Supplementary file 2 — Additional file 2. [file 12913_2022_7941_MOESM2_ESM.docx]

**SUPPLEMENTARY FILE 2: MMAT Critical Appraisal**

|  | **1. QUALITATIVE STUDIES** | | | | | **3. NON-RANDOMIZED STUDIES** | | | | | **4. QUANTITATIVE DESCRIPTIVE STUDIES** | | | | | **5. MIXED METHODS STUDIES** | | | | |
| --- | --- | --- | --- | --- | --- | --- | --- | --- | --- | --- | --- | --- | --- | --- | --- | --- | --- | --- | --- | --- |
| First author, year | 1.1. Is the qualitative approach appropriate to answer the research question? | 1.2. Are the qualitative data collection methods adequate to address the research question? | 1.3. Are the findings adequately derived from the data? | 1.4. Is the interpretation of results sufficiently substantiated by data? | 1.5. Is there coherence between qualitative data sources, collection, analysis and interpretation? | 3.1. Are the participants representative of the target population? | 3.2. Are measurements appropriate regarding both the outcome and intervention (or exposure)? | 3.3. Are there complete outcome data? | 3.4. Are the confounders accounted for in the design and analysis? | 3.5. During the study period, is the intervention administered (or exposure occurred) as intended? | 4.1. Is the sampling strategy relevant to address the research question? | 4.2. Is the sample representative of the target population? | 4.3. Are the measurements appropriate? | 4.4. Is the risk of nonresponse bias low? | 4.5. Is the statistical analysis appropriate to answer the research question? | 5.1. Is there an adequate rationale for using a mixed methods design to address the research question? | 5.2. Are the different components of the study effectively integrated to answer the research question? | 5.3. Are the outputs of the integration of qualitative and quantitative components adequately interpreted? | 5.4. Are divergences and inconsistencies between quantitative and qualitative results adequately addressed? | 5.5. Do the different components of the study adhere to the quality criteria of each tradition of the methods involved? |
| Amaya-Jackson  2018 |  |  |  |  |  | Yes | Yes | Yes | Yes | Yes |  |  |  |  |  |  |  |  |  |  |
| Amaya-Jackson  2018 |  |  |  |  |  | Yes | Yes | Yes | Yes | Yes |  |  |  |  |  |  |  |  |  |  |
| Anaby  2014 | Yes | Yes | Yes | Yes | Yes |  |  |  |  |  |  |  |  |  |  |  |  |  |  |  |
| Anaby  2014 | Yes | Yes | Yes | Yes | Can't tell |  |  |  |  |  |  |  |  |  |  |  |  |  |  |  |
| Beidas  2016 |  |  |  |  |  |  |  |  |  |  |  |  | Yes | No | Yes |  |  |  |  |  |
| Beidas  2016 |  |  |  |  |  |  |  |  |  |  | Yes | Yes | Yes | No | Yes |  |  |  |  |  |
| Couturier  2019 | Yes | Yes | Yes | Yes | Yes |  |  |  |  |  | Yes | Yes | Yes | Yes | Yes | Yes | Yes | Yes | No | Yes |
| Couturier  2019 | Yes | Yes | Yes | Yes | Yes |  |  |  |  |  | Yes | Yes | Yes | Yes | Yes | Yes | Yes | Yes | No | Yes |
| Couturier  2018 | Yes | Yes | Yes | Yes | Yes |  |  |  |  |  |  |  |  |  |  |  |  |  |  |  |
| Couturier  2018 | Yes | Yes | Yes | Yes | Yes |  |  |  |  |  |  |  |  |  |  |  |  |  |  |  |
| Henderson  2017 |  |  |  |  |  |  |  |  |  |  | Yes | Yes | Yes | Yes | Can't tell |  |  |  |  |  |
| Henderson  2017 |  |  |  |  |  |  |  |  |  |  | Yes | Yes | Yes | Yes | Can't tell |  |  |  |  |  |
| Kingsley  2020 |  |  |  |  |  |  |  |  |  |  | Yes | Yes | Yes | Yes | Yes |  |  |  |  |  |
| Kingsley  2020 |  |  |  |  |  |  |  |  |  |  | Yes | Yes | Yes | Yes | Yes |  |  |  |  |  |
| Nadeem  2018 | Yes | Yes | Yes | Yes | Yes | Yes | Yes | Yes | Yes | Yes |  |  |  |  |  | Yes | Yes | Yes | Yes | Yes |
| Nadeem  2018 | Yes | Yes | Yes | Yes | Yes |  |  |  |  |  | Yes | Yes | Yes | Yes | Yes | Yes | Yes | Yes | Yes | Yes |
| Radovic  2019 | Yes | Yes | Yes | Yes | Yes |  |  |  |  |  | Yes | Yes | Yes | Can't tell | Yes | Yes | Yes | No | Can't tell | Yes |
| Radovic  2019 | Yes | Yes | Yes | Yes | Yes |  |  |  |  |  | Yes | Yes | Yes | Can't tell | Yes | Yes | Yes | No | Can't tell | Yes |
| Shafran  2020 | Yes | Yes | Yes | Yes | Yes |  |  |  |  |  |  |  |  |  |  |  |  |  |  |  |
| Shafran  2020 | Yes | Yes | Can't tell | Can't tell | Yes |  |  |  |  |  |  |  |  |  |  |  |  |  |  |  |
| Snider  2016 | Yes | No | Can't tell | No | Can't tell |  |  |  |  |  |  |  |  |  |  |  |  |  |  |  |
| Stanhope  2018 | Yes | Yes | Yes | Yes | Yes |  |  |  |  |  | Yes | Yes | Yes | Yes | Yes | Yes | Yes | Yes | Yes | Yes |
| Stanhope  2018 | Yes | Yes | Yes | Yes | Yes |  |  |  |  |  | Yes | Yes | Yes | Yes | Yes | Yes | Yes | Yes | Yes | Yes |
| Westerlund  2020 | Yes | Yes | Yes | Yes | Yes |  |  |  |  |  |  |  |  |  |  |  |  |  |  |  |
| Westerlund  2020 | Yes | Yes | Yes | Yes | Yes |  |  |  |  |  |  |  |  |  |  |  |  |  |  |  |

* Randomised controlled trial descriptors/variables were inapplicable and have been removed

** Screening questions not included in the table above
